# Child poverty—a political choice

**DOI:** 10.1093/eurpub/ckae138

**Published:** 2024-12-10

**Authors:** Anna Pearce, Alastair H Leyland

**Affiliations:** MRC/CSO Social and Public Health Sciences Unit, University of Glasgow, Glasgow, UK; MRC/CSO Social and Public Health Sciences Unit, University of Glasgow, Glasgow, UK

In the broadest sense, child poverty can be thought of as the absence of a family’s ‘ability to get by and participate in the society in which they live’ [1]. Material poverty is often operationalized as income levels below a threshold relative to a particular context (e.g. a fixed proportion—say 50 or 60%—of a country’s median income) [2]. Within the European Union (EU), levels of child poverty (based on 60% of median income) are over 25% in Romania, Bulgaria, Spain, Italy, and Hungary, and below 10% in Denmark and Slovenia [3]. Income is causally related to children’s physical health and cognitive, social, emotional, and behavioural development, and it impacts on the family environment (including parenting style, home learning environments, and maternal mental health) [4]. Cohort studies have shown the long shadow cast by child poverty, with it affecting academic achievement and employment opportunities, and early and preventable deaths from a range of causes including alcohol-related, cardiovascular disease, and cancer. Poverty and its effects on health can also pass from one generation to the next. Addressing child poverty therefore brings the dual benefits of reducing damage to children who are living in poverty now and large, longer-term gains to the economy [1]. In Europe, the average estimated total cost to society due to childhood socio-economic disadvantage (arising through loss of employment, earnings, and health) amounts to an annual 3.4% of a country’s gross domestic product [5]. This average figure masks considerable variation between the 24 OECD countries studied, ranging from 1.4% of GDP in Finland to 6.1% in Lithuania.

The ability to alter child poverty meaningfully lies in the hands of governments, which have legislative powers that include determining levels of taxation, how revenue is spent, and on whom. In 11 of the 27 EU countries, poverty among children is more common than poverty among those aged 65 and over; indeed, in Luxembourg (17.0%) and France (15.9%), levels of child poverty are more than double the levels seen in the elderly [3]. Governments also choose how to allocate budgets to, e.g. defence, health, and welfare, and how to respond to societal need. Consider e.g. the vast sums of money that were found to support banks and the financial system in the 1990s and 2000s, and the temporary £20 per week uplift in welfare benefits introduced in the UK during the COVID-19 pandemic (this was designed to improve the available support at a time of crisis and lifted 400 000 children out of relative poverty).

A variety of tools are available to governments to alleviate or eradicate child poverty; these include income supplementation through cash transfers or welfare payments, subsidies for some of the largest expenditures faced by parents (e.g. free childcare or school meals), and increasing incomes through paid employment. These approaches may be implemented universally (available to everyone) or targeted (e.g. to those experiencing the highest levels of socio-economic disadvantage). The scope of any targeting may vary; e.g. welfare benefits might be targeted to all children living in low incomes through means-testing, or specifically to low-income families where the parents are teenagers. Another defining feature of child poverty policies, which has potential consequences for health and inequalities, is whether conditions are attached. For example, the receipt of cash transfers may be conditional on engagement with health services (e.g. which may improve immunization uptake) or proof of job searching (which has the potential to increase income but may lead to increased parental stress). Furthermore, if the onus is placed on parents—i.e. if there is a requirement for a claim to be submitted and subsequently approved—then there is the risk that the complexity of the system to be navigated to make a claim will result in the exclusion of some of those in the greatest need. The alternative is to screen for eligibility through the combination of registers (e.g. taxation, welfare) enabling the identification of children living in poverty, with payments triggered automatically.

Although there are short–medium-term financial costs associated with the eradication of child poverty, in the longer term, this is likely to be cost effective. Building a workforce that is healthier and better educated will repay the investment, but the time taken for such a return will be spread across the life course and, most likely, generations. Keeping children in poverty is a political choice influenced by short-term political cycles; it is also the wrong choice for social justice and longer-term prosperity. To quote James Freeman Clarke, ‘A politician… thinks of the next election; while the statesman thinks of the next generation’.

Conflict of interest: None declared.
